# Study participant reported outcomes of mental health interventions: results from a randomized controlled trial among survivors of systematic violence in southern Iraq

**DOI:** 10.1017/gmh.2018.11

**Published:** 2018-05-15

**Authors:** Z. Mahmooth, W. M. Weiss, G. A. S. Zangana, P. Bolton

**Affiliations:** 1 Department of International Health, Johns Hopkins Bloomberg School of Public Health, 615 N. Wolfe Street, Baltimore, MD 21205, USA; 2 Heartland Alliance International, Rizgary Taza 408, Alley 32, House 08, Sulaymaniyah, Iraq

**Keywords:** Global mental health, interventions, Iraq, psychological trauma, randomized controlled trial, torture

## Abstract

**Background.:**

Common mental health problems experienced by survivors of systematic violence include trauma, depression, and anxiety. A trial of mental health interventions by community mental health workers for survivors of systematic violence in southern Iraq showed benefits from two psychotherapies on trauma, depression, anxiety, and function: Common Elements Treatment Approach (CETA) and cognitive processing therapy (CPT). This study assessed whether other non-predetermined changes reported by intervention participants were more common than in the control group.

**Methods.:**

The trial involved 342 participants (CETA: 99 intervention, 50 control; CPT: 129 intervention, 64 control). Sixteen intervention-related changes since enrollment were identified from free-listing interviews of 15 early therapy completers. The changes were then added as a new quantitative module to the follow-up questionnaire. The changes were organized into eight groupings by thematic analysis – family, social standing, anger management, interest in regular activities, optimism, feeling close to God, avoiding smoking and drugs, and physical health. All participants were interviewed with this module and responses were compared between intervention and control participants.

**Results.:**

Multi-level, multi-variate regression models showed CETA intervention subjects with significant, positive changes relative to CETA controls on most themes. CPT intervention subjects showed little to no change compared with CPT controls in most themes.

**Conclusions.:**

Participants receiving CETA reported more positive changes from therapy compared with controls than did participants receiving CPT. This study suggests differential effects of psychotherapy beyond the predetermined clinical outcome measures and that identification of these effects should be part of intervention evaluations.

## Introduction

Psychotherapeutic interventions have been studied for their effectiveness by assessing improvement in outcomes related to symptoms of psychological disorders, such as post-traumatic stress disorder (PTSD), depression, and anxiety, resulting from torture (Weiss *et al.*, 2016). The implementation of interventions themselves may be associated with changes that were not specifically intended or expected to the participants as well as their families, the healthcare providers, and communities. Bolton *et al*., have called for expanding the scope of evaluations to also explore the possibility of these changes as outcomes that were not determined *a priori* but reported and determined by participants themselves (Bolton *et al.*, 2007). They argue that these changes might be of more importance than the primary goals in terms of their impact on intervention recipients and the wider population. Substantial negative unexpected changes could explain why programs are not successful. They could even lead to the conclusion that the intervention has done more harm than good, even if the predetermined goals have been achieved. Possible unexpected changes, by definition, are not known prior to implementation of the trial and therefore not measured at baseline. The purpose of this study was, therefore, to first compile a list of participant-reported changes due to the interventions from a subset of early therapy completers and then quantitatively assess if these changes were more significant in the intervention groups compared with the controls.

The study is part of a larger research effort of psychotherapeutic interventions by community mental health workers (CMHWs) in Iraq. Trials were conducted using the same study protocol in different regions of Iraq. A randomized controlled trial of behavioral activation treatment for depression (BATD) and cognitive processing therapy (CPT) delivered by CMHWs was tested in the Iraqi Kurdistan cities of Sulaymaniyah and Erbil. The results of that trial showed both therapies providing an improvement in dysfunction symptoms and BADT possibly providing an improvement in depression symptoms as well (Bolton *et al.*, 2014). A randomized trial of a counseling intervention by CMHWs in the Dohuk region of Iraqi Kurdistan showed significant improvements in the treated population for depression and dysfunction symptoms (Bass *et al.*, 2016). The behavior changes reported by study participants analyzed in this paper are from two concurrently conducted mental health interventions for torture survivors in the south of Iraq, which has not enjoyed the relative stability of Iraqi Kurdistan. The interventions were conducted in two areas in the south of Iraq with each area receiving a different intervention. The interventions were conducted in existing health facilities where local Heartland Alliance staff and the Ministry of Health were already providing non-specific counseling. CPT was used in Basra and its surrounding areas. Common Elements Treatment Approach (CETA), a new intervention recently developed by Johns Hopkins University and University of Washington faculty with input from the local supervisors and providers who would deliver the therapy, was used in Karbala and its surrounding areas.

CETA is designed to be flexible and personalized to the individual client. Each client's treatment consists of different therapeutic components that are deemed relevant to the individual's symptoms. Components consist of encouraging participation, psycho-education, cognitive coping, gradual exposure by discussion, live exposure to feared places or things, cognitive reprocessing, safety skills, and behavioral coping techniques. Sessions last approximately an hour and are conducted weekly from 10 to 12 weeks. Weekly homework is also included.

CPT was used in an earlier, similar study in Iraqi Kurdistan (Bolton *et al.*, 2014). CPT was developed to treat PTSD symptoms in rape victims. CPT deals with clients’ extreme or unhelpful thoughts due to a traumatic event and attempts to help clients develop more helpful thoughts, beliefs, and behaviors. It does this by attempting to have the client identify exaggerated beliefs and behaviors stemming from the trauma by remembering and processing them into more balanced beliefs and behaviors. CPT consists of 12 weekly sessions and also includes homework. Under the supervision of clinical psychiatrists, CMHWs were trained in and delivered either the CETA or CPT intervention, depending on their location.

The CETA intervention showed large improvements in the primary outcomes of trauma, depression, anxiety, and dysfunction symptoms in the intervention group and minimal change among the controls. The results from the CPT intervention showed moderate improvements in the primary outcomes of trauma and depression symptoms compared with the controls. However, unlike the CETA controls, the CPT controls showed more improvement, so that there was no statistically significant difference in improvement in anxiety and dysfunction symptoms between CPT intervention and control participants. The complete details of the interventions and results of the primary outcomes of the trial are available in an earlier publication (Weiss *et al.*, 2015). The results of the participant reported changes due to the interventions are the focus of this paper.

## Methods

The trial was approved by the Institutional Review Board at the Johns Hopkins Bloomberg School of Public Health and by the Ministry of Health of Iraq. This trial was registered at ClinicalTrials.gov with identifier NCT01177072.

### Setting

The study took place in southern Iraq with a CETA intervention and control arm in Karbala, Najaf, Hilla, and the surrounding areas and a CPT intervention and control arm in Basra, Nassariyah, and the surrounding areas. The population has significant numbers of torture survivors from the Saddam Hussein era in the 1970s, through the US invasion in 2003, and up till the instability today (Korn & Human Rights Watch, 1990; Human Rights Watch, 1993; Amnesty International, 2013). Political prisoners, their family members, convicts, and other detained persons have faced systemic violence due to lack of accountability and the high value placed on confessions of prisoners in Iraqi courts (Amnesty International, 2013; Human Rights Watch, 2013; UN Assistance Mission for Iraq, 2013). Therapy was provided by CMHWs in Ministry of Health primary healthcare centers or, if distance or privacy was a concern, a location mutually agreed upon by both CMHW and participant.

### Participants

The participants of this study on unexpected changes were the same as that of the randomized controlled trial looking at the primary outcomes of trauma, anxiety, and depression. Eligible participants for the study were adults living in southern Iraq who scored higher than 35 on the CMHW administered intake instrument and were affected by torture. The threshold intake score was determined by a validity study of the intake instrument with a clinical diagnosis of PTSD. Torture experience included being personally subjected to torture and imprisonment, witnessing torture, or having a close family member as a torture survivor. Exclusion criteria included those who were deemed in danger of committing suicide and those deemed mentally incompetent to give consent to participating in the study. Eligible persons for screening were found largely through prisoner associations, a torture rehabilitation and treatment center in Basra, and also through identifying participants when they presented to health clinics seeking treatment. Intervention to control was allocated on a 2:1 ratio at both sites. Control participants were on a waitlist period and received monthly telephone calls from CMHWs to assess if they were safe and if they needed emergent psychiatric care referral. After the follow-up assessment at the end of the control period, they were offered CETA or CPT.

### Measures

**Fig. 1.**
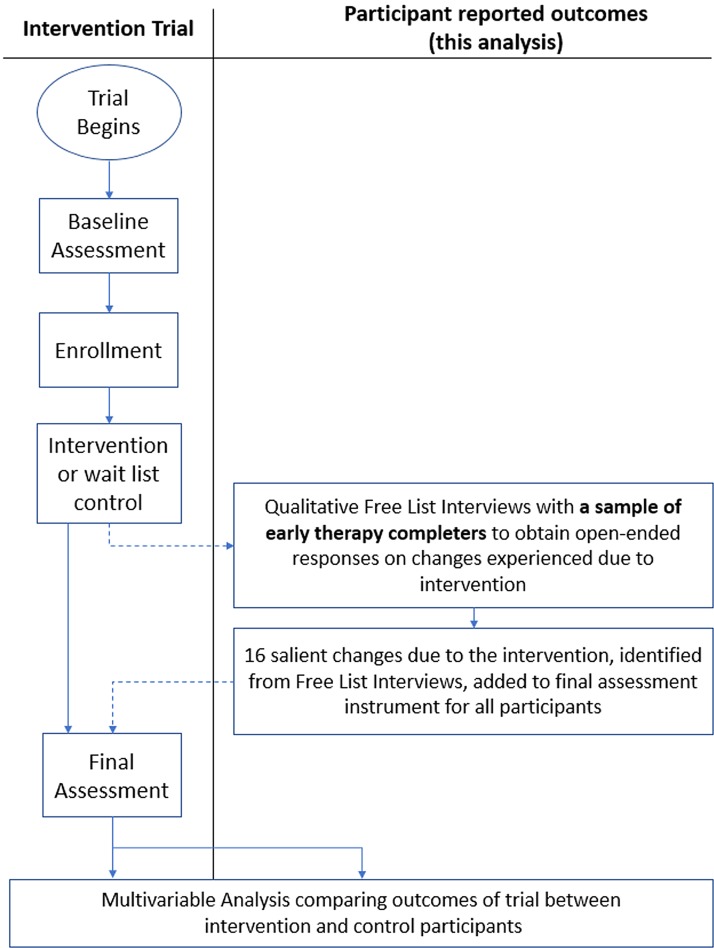
Study design with a qualitative free-listing interview to identify salient changes due to the intervention that are incorporated into follow-up assessment as a quantitative module for final analysis.

The first group of study participants to complete CETA and CPT were interviewed in individual free-listing sessions. These sessions are open-ended qualitative interviews. They were asked what were all the changes they or their family have experienced since beginning the program and also changes they experienced because of the program. From the combined responses in both CETA and CPT arms, 16 salient changes due to the interventions were identified by the frequency of that response across interviews. These 16 items were then added as an additional module to the follow-up instruments for intervention and control participants (Fig. 1). For each question, study participants were asked to ‘compare how things are for (them) to how they were several months ago when they were first interviewed’ (i.e., prior to treatment) in relation to one of the 16 possible changes (e.g., ‘Avoiding smoking cigarettes or using drugs’).

### Data collection

After determination of intervention-related change items from early therapy completers, follow up for intervention participants was conducted after at least 90 days had passed from the completion of therapy. Early therapy completers who participated in the free-listing session to construct the change items were also included in the complete follow-up assessment and assessed on those items. Within 1 to 2 weeks of when an intervention participant was scheduled for follow up, a control participant, who started the wait period about the same date, was assessed for follow up. A different CMHW who was blind to the treatment assignment of the participant would conduct the follow-up assessment at the end of the treatment for intervention participants or the wait period for controls. The follow-up assessment was similar to the intake assessment except for the inclusion the 16 questions about intervention-related changes observed since intake into the study.

### Analysis

Statistical analysis was conducted in Stata 12. Multiple imputations were used to generate missing values for each of the 16 participants reported changes at the response level for any missing follow-ups. Missing scores were calculated using available demographic information and changes reported by other participants using predictive mean matching to generate 11 multiple imputation datasets. The imputation was run separately for CETA and CPT participants.

**Fig. 2.**
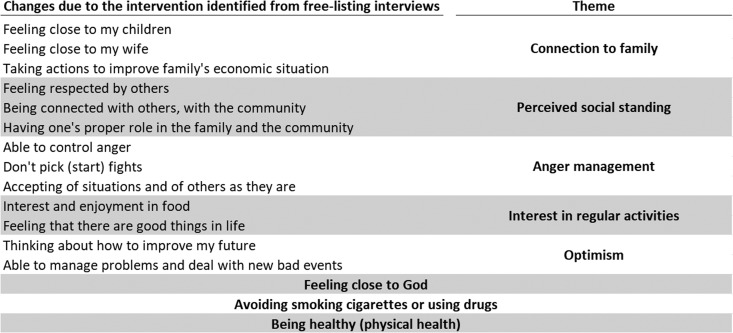
Thematic groupings of participant-reported changes due to the intervention.

The 16 participant reported changes were analyzed independently or grouped together by an overlying theme (Fig. 2). The items grouped under eight themes were averaged to provide the value for that theme. Responses were analyzed in the data as scores from −2 to 2, with −2 corresponding to ‘much worse’, 0 to ‘no change’, and 2 to ‘much better.’ Multi-level, multi-variate regression models were used to determine if any of the eight change themes were experienced to a significantly larger or lesser degree by intervention *v.* control participants. To isolate the effect of the CETA and CPT, further analysis was done to identify covariates or interactions requiring an adjustment in the final model. Gender, age, employment, and marriage were chosen *a priori* to be included as covariates in the outcome models regardless of statistical significance. Any demographic differences between intervention and control participants that were significant at *p* < 0.5 were included as covariates in the models. The significance of any additional adjustments was determined by statistical significance (*p* < 0.05) of the adjustment coefficient and likelihood-ratio tests between the parsimonious model and the model extended with the additional adjustment. Finally, Cohen's d effect sizes of the intervention on possible unexpected changes were calculated by dividing the treatment coefficient, which represented the difference in means between intervention and control, by the standard deviation of the unadjusted mean outcome. A Cohen's d in the 0.2–0.5 range was interpreted as a small intervention effect, 0.51–0.8 as a moderate intervention effect, and > 0.8 as a large intervention effect.

## Results

**Fig. 3.**
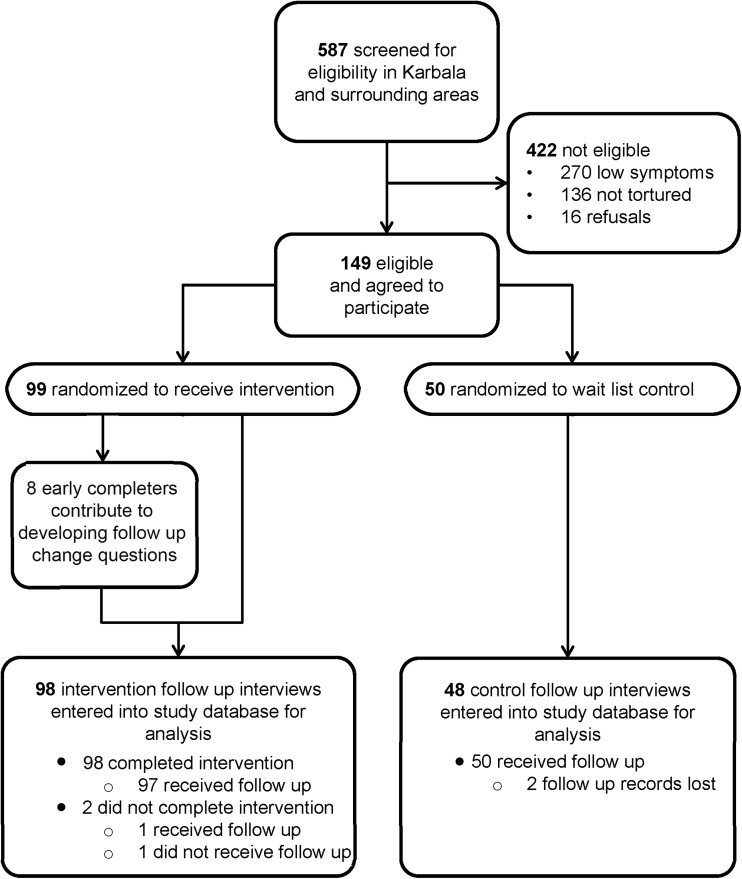
Participant flowchart for CETA intervention and control arms.

Five-hundred and eighty-seven persons were screened for study eligibility in Karbala and its surrounding areas for entry into the CETA intervention and control arms, and 422 were found ineligible for the study (Fig. 3). Among those screened, approximately a third had not experienced torture. An additional 16 persons who met eligibility criteria refused to participate. Of the 149 who were then enrolled in the study, 99 were allocated to the CETA intervention arm and 50 were allocated to the 5-month wait-control arm after randomization. From the CETA intervention arm, eight participants participated in a free-listing interview to develop the change questions. Only two persons did not complete the therapy, one of whom could not be reached for follow up. All 97 persons who completed therapy received follow up. All 50 wait-list controls were reached for follow up, but the follow-up forms were subsequently lost for two of them.

**Fig. 4.**
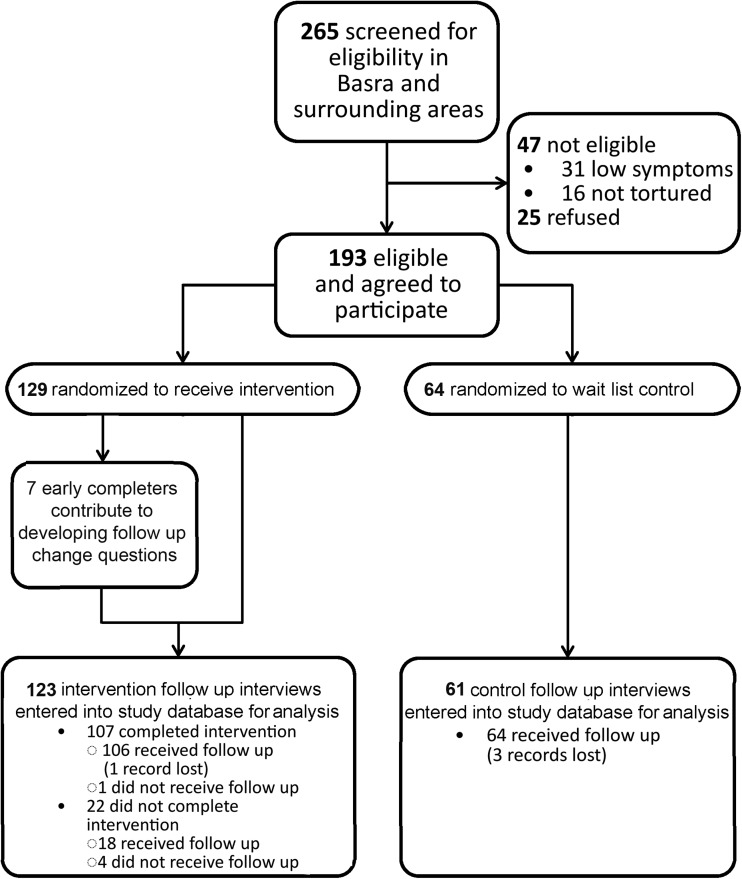
Participant flowchart for CPT intervention and control arms.

Similarly, 265 persons were screened for the CPT intervention and control arms in Basra and its surrounding area (Fig. 4). Forty-seven persons did not meet eligibility, with about a third due to not experiencing torture. An additional 25 persons who met eligibility criteria refused to participate. Of the 193 who were then enrolled in the study, 129 were allocated to the CPT intervention arm and 64 were allocated to the 5-month wait-control arm after randomization. From the CPT intervention arm, seven participants participated in a free-listing interview to develop the change questions. Twenty-two persons did not complete therapy, four of whom could not be reached for follow up. One person who completed therapy could not be reached for follow up and the follow-up form for one person who completed therapy and follow up was lost. All 64 wait-list controls received follow up, but the follow-up paperwork was unable to be located for three of them.

CETA intervention and CETA controls differed in terms of the proportion married, those with a disability, and those with a bachelor's degree or higher (Table 1). There were no significant differences between CPT intervention and CPT controls at baseline. A total of 12 participants who had missing follow-up records, three in CETA and nine in CPT, had their change scores generated by multiple imputations. Data from a total of 99 CETA intervention, 50 CETA control, 129 CPT intervention, and 64 CPT control participants were included in the final intent-to-treat analysis.

**Table 1.** Baseline characteristics of intervention and control participants

	CETA		CPT	
	Intervention	Controls	Intervention	Controls
Size	99	50	129	64
Sex				
Male, *N* (%)	67 (67.7)	36 (72)	87 (67.4)	40 (62.5)
Female, *N* (%)	32 (32.3)	14 (28)	42 (32.6)	24 (37.5)
Age				
Mean (SD)	41.55 (11.3)	45.16 (11.1)	39.73 (12.3)	41.02 (9.5)
Children				
Mean (SD)	2.30 (2.0)	2.34 (2.0)	2.17 (2.1)	2.66 (2.1)
Marital status				
Single, *N* (%)	13 (13.1)	2 (4.0)	20 (15.5)	4 (6.3)
Married, *N* (%)	73 (73.7)^a^	44 (88.0)^a^	95 (73.6)	50 (78.1)
Widowed, *N* (%)	10 (10.1)	3 (6.0)	4 (3.1)	4 (6.3)
Divorced, *N* (%)	3 (3.0)	1 (2.0)	10 (7.8)	6 (9.4)
Working status				
Not working, *N* (%)	36 (36.4)	17 (34.0)	55 (42.6)	24 (37.5)
Irregular or daily, *N* (%)	25 (25.3)	8 (16.0)	19 (14.7)	11 (17.2)
Regular or stable, *N* (%)	34 (34.3)	20 (40.0)	46 (35.7)	25 (39.1)
Self-employed, *N* (%)	4 (4.0)	5 (10.0)	9 (7.0)	4 (6.3)
Education				
None, *N* (%)	15 (15.0)	3 (6.0)	20 (15.5)	13 (20.3)
Primary, *N* (%)	30 (30.3)	21 (42.0)	48 (37.2)	32 (5)
Secondary, *N* (%)	33 (33.3)	12 (24.0)	29 (22.5)	12 (18.8)
Institutional degree, *N* (%)	16 (16.2)	6 (12.0)	18 (14.0)	4 (6.3)
Bachelor's or higher, *N* (%)	5 (5.1)^a^	8 (16.0)^a^	14 (10.9)	3 (4.7)
Disability				
Yes, *N* (%)	13 (13.1)^a^	1 (2.0)^a^	9 (7.0)	5 (7.8)
No, *N* (%)	86 (86.9)^a^	49 (98.0)^a^	120 (93.0)	59 (92.2)

a Significant difference (*p* < 0.05) between CETA interventions and CETA controls.

### CETA outcomes

Large positive therapeutic effects were observed in the themes of family connections, perceived social standing, anger management, interest in regular activities, and optimism among CETA intervention participants in comparison with CETA controls (Table 2). CETA intervention participants reported significant improvements in these intervention-related changes. In contrast, CETA control participants reported improvement only in the change themes of interest in regular activities and optimism, and then at a significantly lower level of change compared with intervention participants. Both intervention and control participants reported some improvement in ‘Feeling close to God’ but there was no significant difference between intervention and control participants. For this outcome, therapy participants who had children reported more improvement than therapy participants with no children as suggested by the significant interaction between intervention status and having children covariate (*β*  =  0.91, *p*  =  0.045). Medium positive therapeutic effects were observed in the mean response to non-categorized possible changes of ‘Avoiding smoking cigarettes or using drugs,’ and ‘Being healthy (physical health).’

**Table 2.** Difference in mean change theme scores comparing CETA intervention with CETA control participants

	CETA Intervention (*N* = 99)		CETA Controls (*N* = 50)		Net effect*		Effect size
	Score	95% CI	Score	95% CI	Score	95% CI	Cohen's d
Connection to family^a^^,^^b^	1.09	0.71–1.47	0.34	−0.07–0.75	0.75	0.35–1.15	0.86
Perceived social standing^c^^,^^b^	1.03	0.73–1.34	0.29	−0.10–0.68	0.74	0.37–1.12	0.91
Anger management^d^^,^^b^	1.13	0.83–1.42	0.25	−0.15–0.65	0.87	0.48–1.26	1.09
Interest in regular activities^e^^,^^b^	1.05	0.71–1.38	0.40	0.01–0.79	0.65	0.33–0.96	0.80
Optimism^f^^,^^b^	1.07	0.77–1.37	0.38	0.04–0.72	0.69	0.37–1.01	0.89
Feeling close to God^g^	0.74	0.24–1.24	1.16	0.51–1.81	−0.42	−1.22–0.38	−0.42
Avoiding smoking cigarettes or using drugs^b^	0.77	0.29–1.26	0.19	−0.34–0.72	0.58	0.10–1.07	0.55
Being healthy (physical health)^b^	1.08	0.67–1.49	0.46	−0.05–0.96	0.62	0.15–1.10	0.61

a Connection to family items consist of ‘Feeling close to my children,’ ‘Feeling close to my wife,’ and ‘Taking actions to improve family's economic situation.

b Adjusted for gender, age, unemployment, marriage, and disability.

c Perceived social standing items consist of ‘Feeling respected by others,’ ‘Being connected with others, with the community,’ and ‘Having one's proper role in the family and the community.’

d Anger management items consist of ‘Able to control anger,’ ‘Don't pick (start) fights,’ and ‘Accepting of situations and of others as they are.’

e Interest in regular activities items consist of ‘Interest and enjoyment in food,’ and ‘Feeling that there are good things in life.’

f Optimism items consist of ‘Able to manage problems and deal with new bad events,’ and ‘Thinking about how to improve my future.’ The model-estimated difference at post-test, adjusting for baseline differences, including CMHW as a cluster variable, and other factors indicated below.

g Adjusted for gender, age, unemployment, marriage, disability, and having children and its interaction with treatment.

### CPT outcomes

Both CPT intervention participants and CPT controls showed large and statistically significant improvement on all categories of possible unexpected changes (Table 3). There was a medium therapy effect for the optimism themed changes. On all other possible change themes, there was no significant effect of therapy compared with the controls. Significant interaction terms between education and intervention status suggested that CPT intervention participants who had no formal education had less improvement in the optimism themed changes than those receiving CPT who had a primary or higher level education (*β*  =  −0.73, *p*  =  0.032); the same observation was found for the non-category change about ‘avoiding cigarettes and other drugs’ (*β*  =  −0.87, *p*  =  0.033).

**Table 3.** Difference in mean change theme scores comparing CPT intervention with CPT control participants

	CPT Intervention (*N* = 129)		CPT Controls (*N* = 64)		Net effect*		Effect size
	Score	95% CI	Score	95% CI	Score	95% CI	Cohen's d
Connection to family^a^^,^^b^	1.12	0.95–1.29	0.92	0.67–1.12	0.20	0.00–0.39	0.5
Perceived social standing^c^^,^^b^	1.00	0.87–1.13	0.90	0.70–1.11	0.10	−0.10–0.29	0.19
Anger management^d^^,^^b^	1.00	0.86–1.14	0.85	0.68–1.03	0.15	0.00–0.30	0.32
Interest in regular activities^e^^,^^b^	1.04	0.86–1.22	0.91	0.73–1.09	0.13	−0.08–0.33	0.22
Optimism^f^^,^^g^	1.11	0.96–1.26	0.78	0.59–0.98	0.32	0.16–0.48	0.55
Feeling close to God^b^	1.29	1.10–1.48	1.32	1.10–1.54	−0.03	−0.22–0.17	−0.04
Avoiding smoking cigarettes or using drugs^g^	0.96	0.61–1.31	0.81	0.40–1.22	0.15	−0.25–0.54	0.17
Being healthy (physical health)^b^	1.10	0.95–1.24	0.89	0.61–1.17	0.21	−0.06–0.47	0.28

a Connection to family items consist of ‘Feeling close to my children,’ ‘Feeling close to my wife,’ and ‘Taking actions to improve family's economic situation.

b Adjusted for gender, age, unemployment, and marriage.

c Perceived social standing items consist of ‘Feeling respected by others,’ ‘Being connected with others, with the community,’ and ‘Having one's proper role in the family and the community.’

d Anger management items consist of ‘Able to control anger,’ ‘Don't pick (start) fights,’ and ‘Accepting of situations and of others as they are.’

e Interest in regular activities items consist of ‘Interest and enjoyment in food,’ and ‘Feeling that there are good things in life.’

f Optimism items consist of ‘Able to manage problems and deal with new bad events,’ and ‘Thinking about how to improve my future.’ The model-estimated difference at post-test, adjusting for baseline differences, including CMHW as a cluster variable, and other factors indicated below.

g Adjusted for gender, age, unemployment, marriage, and education and its interaction with treatment.

## Discussion

The evaluation of mental health interventions should attempt to identify and measure changes, including those outside of predetermined measures that may be unexpected or unintended, so as to gain a fuller picture of the beneficial and harmful effects of interventions. These changes reveal often overlooked but potentially important effects of mental health interventions in several areas.

The primary study measured clinical outcomes such as trauma, anxiety, and depression but participants described other outcomes that were important to them and which primarily referred to family and social relationships.

We found a similar pattern for these other outcomes as for the study primary outcomes: While both CETA and CPT participants showed substantial improvements, improvement in the CETA controls was minimal while that among the CPT controls was comparable with the CPT intervention group. This resulted in statistically significant differences in the CETA intervention compared with the controls but no statistically significant difference in the level of benefit for CPT intervention group as compared with CPT controls, except for the optimism themed changes. Given the differences in the two sites (the CETA site had more ongoing violence and threat), this suggests that therapy was beneficial in countering the impact of ongoing insecurity and violence for both predetermined and other outcomes but may not be as beneficial in less challenging circumstances.

### Connection to family, perceived social standing

Of the 16 intervention-related change items identified from the free-listing interviews, three were about the connection to family and three were about participant's social standing. This suggests that for persons with mood and trauma disorders, social and inter-personal functioning are important and should be included in assessment (Vittengl *et al.*, 2004; Charuvastra & Cloitre, 2008). Interventions should therefore also consider the secondary impacts to the family and community when evaluating treatment benefits.

### Anger management, interest in regular activities

Evaluation of items related to study participant's management of anger and interest in regular activities are not dissimilar from what we expect the evaluation of primary outcomes such as anxiety and depression to reveal. In this case, the free-listing interviews highlighted items that were similar to the predetermined outcomes and part of the expected changes from implementing the interventions.

### Optimism

Optimism was the only significant change identified from the free-listing interviews that were statistically significantly improved in CPT intervention participants compared with the controls. This is because CPT controls showed the least improvement in this outcome. If CPT was solely evaluated on the primary outcomes of trauma, anxiety, depression, and dysfunction it would only show moderate to no effect. But this analysis revealed that participants in CPT were benefiting from the intervention in that the intervention appeared to give them some hope for future improvement, which could be important for long-term continuation in seeking treatment. More formal education (defined as completing schooling at the primary level or higher) prior to beginning therapy may allow participants to derive a greater benefit from the therapy as suggested by its significant interaction with this outcome in CPT participants. It is possible, however, that participants who had a formal education may simply be more optimistic about the future because of the practical benefits of a formal education.

### Feeling close to God

There was no religious or spiritual component to either CETA or CPT interventions but this was a change identified as important in the intervention participants. When assessed on this change, CETA participants reported a more beneficial change in ‘feeling closer to God’ if they had children *v.* if they had none. It may be possible that participants who had children are more invested in improving themselves through therapy for their family or having children encourages them to be more religious, but why specifically having children helped CETA participants in ‘feeling closer to God’ and no other outcomes is unclear.

### Avoiding smoking cigarettes or other drugs, being healthy

There was no specific substance abuse component of CETA or CPT interventions but it was a change identified as important in the intervention participants. Having any formal education was significantly beneficial in helping CPT participants derive benefit from therapy in avoiding substance use. Those in the CPT trial arm may also have been less predisposed to smoking and drug use prior to beginning the intervention as education was significantly beneficial regardless of whether a subject was an intervention or control participant.

### Strengths and limitations

An inherent limitation of this study is that the questions about changes due to the intervention were identified and measured after the study had begun and after the first cohort of study subjects in the intervention arm completed therapy. Therefore the evidence generated from this study can only be suggestive. In addition, as with the main study, this study also shares the limitations of the primary study in that the follow-up assessment was done, on average, four months after completion of therapy. Therefore we do not know if any of the therapeutic effects are sustained in the long term. The differences in the populations at the two separate study sites did not allow for a direct comparison of the two therapies. Interventions can also cause unexpected changes to participant's family members and other connections in the community, including the healthcare workers providing the intervention. This study limited assessment of unexpected changes to the participants themselves but would have benefited from expanding the assessment to include those populations as well.

We found many changes that CETA and CPT participants reported as important effects of the interventions that were not among the predetermined outcomes of the trial. This is an important practice to ensure that there was no unanticipated harm that resulted and to understand what aspects of implementing the intervention the participants found valuable. Importantly, these outcomes are shaped by the participants themselves rather than what might have been solely of interest to the investigators. The changes include non-clinical outcomes that are of social, economic, and general health interest. Based on the number and diversity of these effects, we suggest that the identification and measurement of broader psychosocial benefits and harms should form part of future assessments of the impacts and utility of these types of interventions.
